# Downfolding of the epiglottis into the laryngeal inlet after tracheal intubation using the McGRATH^TM^ MAC videolaryngoscope: a case report

**DOI:** 10.1186/s40981-020-00349-0

**Published:** 2020-06-05

**Authors:** Haruno Soma, Kenta Furutani, Ayaka Hibino, Akinobu Hibino, Hiroshi Baba

**Affiliations:** 1 Department of Anesthesiology, Niigata University Medical and Dental Hospital, 1-754 Asahimachi-dori, Chuo-ku, Niigata, 951-8520 Japan; 2 Department of Anesthesiology, Niigata City General Hospital, 463-7 Shumoku, Chuo-ku, Niigata, 951-8550 Japan

**Keywords:** McGRATH^TM^ MAC, Videolaryngoscope, Tracheal intubation, Epiglottic downfolding, Epiglottis, Laryngeal inlet

## Abstract

**Background:**

Downfolding of the epiglottis into the laryngeal inlet is considered to be a rare complication of tracheal intubation. We describe a case of epiglottic downfolding during tracheal intubation using a McGrath videolaryngoscope (McGRATH^TM^ MAC).

**Case presentation:**

A 44-year-old female was scheduled for breast reconstruction surgery. Intubation was performed using a McGrath videolaryngoscope. After intubation, videolaryngoscopy revealed that the epiglottis was inverted and folded down into the laryngeal inlet. We elevated the larynx anteriorly using the McGrath videolaryngoscope, enabling the downfolded epiglottis to be pulled out from the laryngeal inlet and restored to its original position. After surgery, the patient was extubated without any complications.

**Conclusions:**

When using the McGrath videolaryngoscope, both glottic exposure similar to that achieved with the Macintosh laryngoscope and careful observation of the epiglottis should enable the prevention, detection, and treatment of epiglottic downfolding into the laryngeal inlet.

## Background

Downfolding of the epiglottis into the laryngeal inlet is a rare complication of tracheal intubation. Prolonged downfolding may result in swelling of the epiglottis after extubation although no reports have described severe airway obstruction caused by epiglottic downfolding.

Epiglottic downfolding into the laryngeal inlet has been reported during or after tracheal intubation using the Macintosh laryngoscope [1, 2], the intubating laryngeal mask airway [3], a fiberscope [4], and lighted stylets [5, 6]. Downfolding of the epiglottis has also been reported when using a rigid indirect videolaryngoscope, the Pentax Airway Scope (Pentax-AWS®; Hoya Corporation, Tokyo, Japan) [7]. Two cases of epiglottis malposition were identified in this report; the authors suggest that the prolapse may not have been visible on the Pentax-AWS screen because the positioning of the camera means that the tip of the blade can impede the view of the epiglottis.

The McGrath videolaryngoscope (McGRATH^TM^ MAC; Airclaft Medical Ltd, Edinburgh, UK) has been widely used both for patients with normal airways and those who are difficult to intubate. Although the manufacturer’s recommendation is that the tip of the blade should be placed in the vallecula, as for the Macintosh laryngoscope, it is also possible to elevate the epiglottis directly if necessary. To our knowledge, there are currently no published reports of the epiglottis downfolding into the laryngeal inlet when using the McGrath videolaryngoscope. Here, we describe a case of epiglottic downfolding during tracheal intubation using the McGrath videolaryngoscope.

## Case presentation

Written informed consent was obtained from the patient for publication of this case report and accompanying images. In our institution, case reports are exempted from IRB approval.

A 44-year-old female (height 151.5 cm, weight 49.5 kg) was scheduled for breast reconstruction surgery using a pedicled flap of the latissimus dorsi muscle. Although the patient did not present any complication in her airway during the preoperative evaluation, the anesthesia record of her previous mastectomy procedure stated that tracheal intubation was performed using a McGrath videolaryngoscope due to difficulties using a Macintosh laryngoscope. Therefore, we planned to use a McGrath videolaryngoscope as the first choice to enable a smooth intubation.

**Fig. 1:**
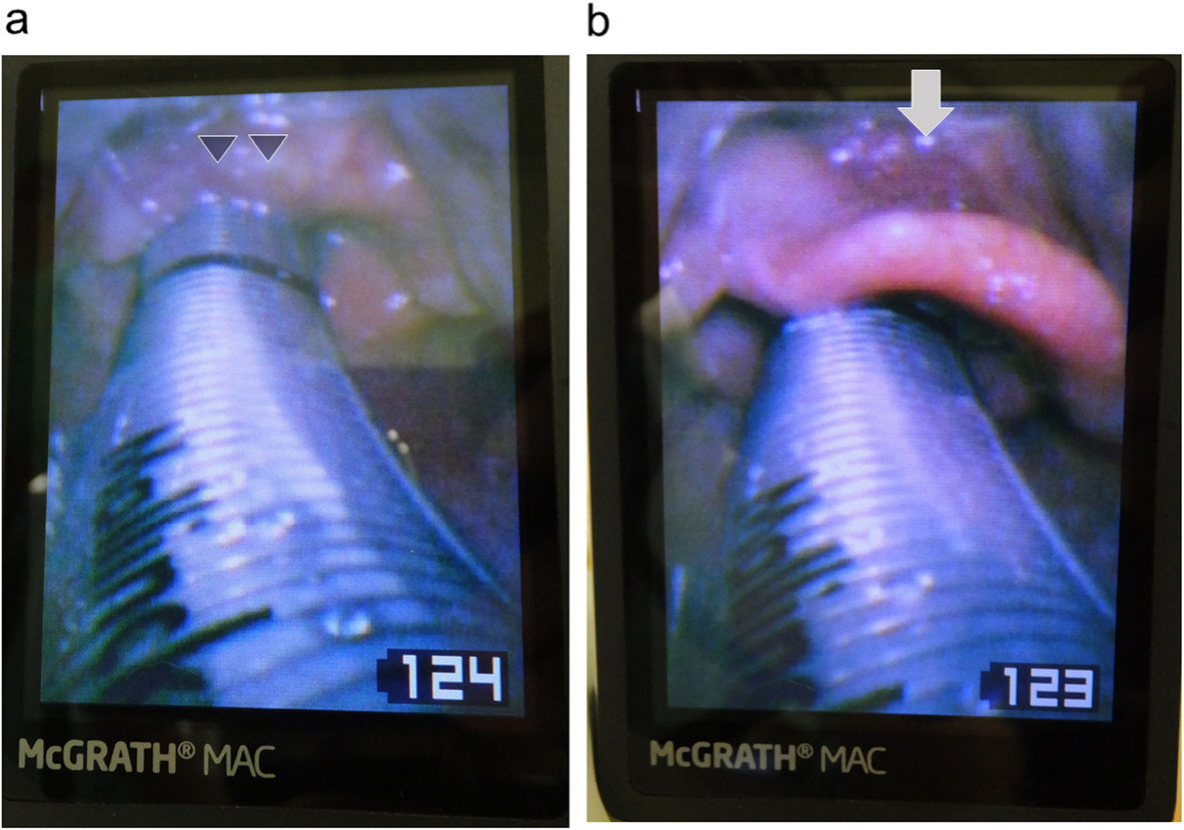
Views of the larynx as seen on the screen of the McGrath videolaryngoscope. **a** After tracheal intubation, the epiglottis (arrowheads) is prolapsed and folded down into the laryngeal inlet. **b** The epiglottis has been restored to its original position by gentle glottic exposure with the McGrath videolaryngoscope blade. Only slight submucosal hemorrhage can be observed on the surface of the epiglottis (arrow), and swelling has not occurred

General anesthesia was induced and maintained with a continuous infusion of propofol (3.0 μg/ml, target controlled infusion) combined with remifentanil (0.4 μg/kg/min). Rocuronium bromide (40 mg) was administered intravenously to facilitate tracheal intubation. Neuromuscular monitoring was not used because it was not available at that time. After mask ventilation, intubation was easily performed by an anesthesia resident using a McGrath videolaryngoscope. The resident had experienced approximately 10 cases of tracheal intubation using this device. However, the supervising anesthesiologist who specialized in difficult airway management suspected that the epiglottis had been inverted by the blade of the McGrath videolaryngoscope and pushed into the laryngeal inlet. After fixation of the endotracheal tube, the supervising anesthesiologist attempted to gain a detailed view of the condition of the epiglottis using McGrath videolaryngoscopy. It was established that the epiglottis was prolapsed; it had been pushed into the laryngeal inlet along with the endotracheal tube (Fig. 1a).

Although we believed that the problem would be easily solved by extubation, we discussed another solution because her subsequent intubation might have proved to be difficult even though the initial intubation had been easy to achieve. Firstly, we attempted to elevate the larynx in an anterior direction using the McGrath videolaryngoscope aiming to restore the epiglottis to its normal position by widening the laryngeal space. We inserted the tip of the McGrath blade carefully into the vallecula. When the larynx was elevated anteriorly, we were fortunately able to pull the downfolded epiglottis out from the laryngeal inlet and restored it to its original position (Fig. 1b). We observed only a little submucosal hemorrhage on the surface of the epiglottis and no swelling. Approximately 8 h after the surgery, the patient was extubated smoothly without respiratory symptoms. No postoperative complications were observed except for slight hoarseness.

## Discussion

This case report suggests that downfolding of the epiglottis into the laryngeal inlet can occur during tracheal intubation when using the McGrath videolaryngoscope and that the downfolded epiglottis can be restored to its original position by gentle glottic exposure using the McGrath blade. Epiglottic downfolding into the laryngeal inlet has been considered to be a rare complication of tracheal intubation [7]. However, as anesthesiologists rarely observe the larynx after tracheal intubation, this prolapse may occur much more frequently than it was currently thought. Although there are fortunately no reports of severe complications associated with the downfolding of the epiglottis, prolonged prolapse of the epiglottis may cause swelling, resulting in airway obstruction [1]. Therefore, if possible, the position of the epiglottis should be carefully observed during tracheal intubation to reduce the risk of prolonged prolapse.

**Fig. 2:**
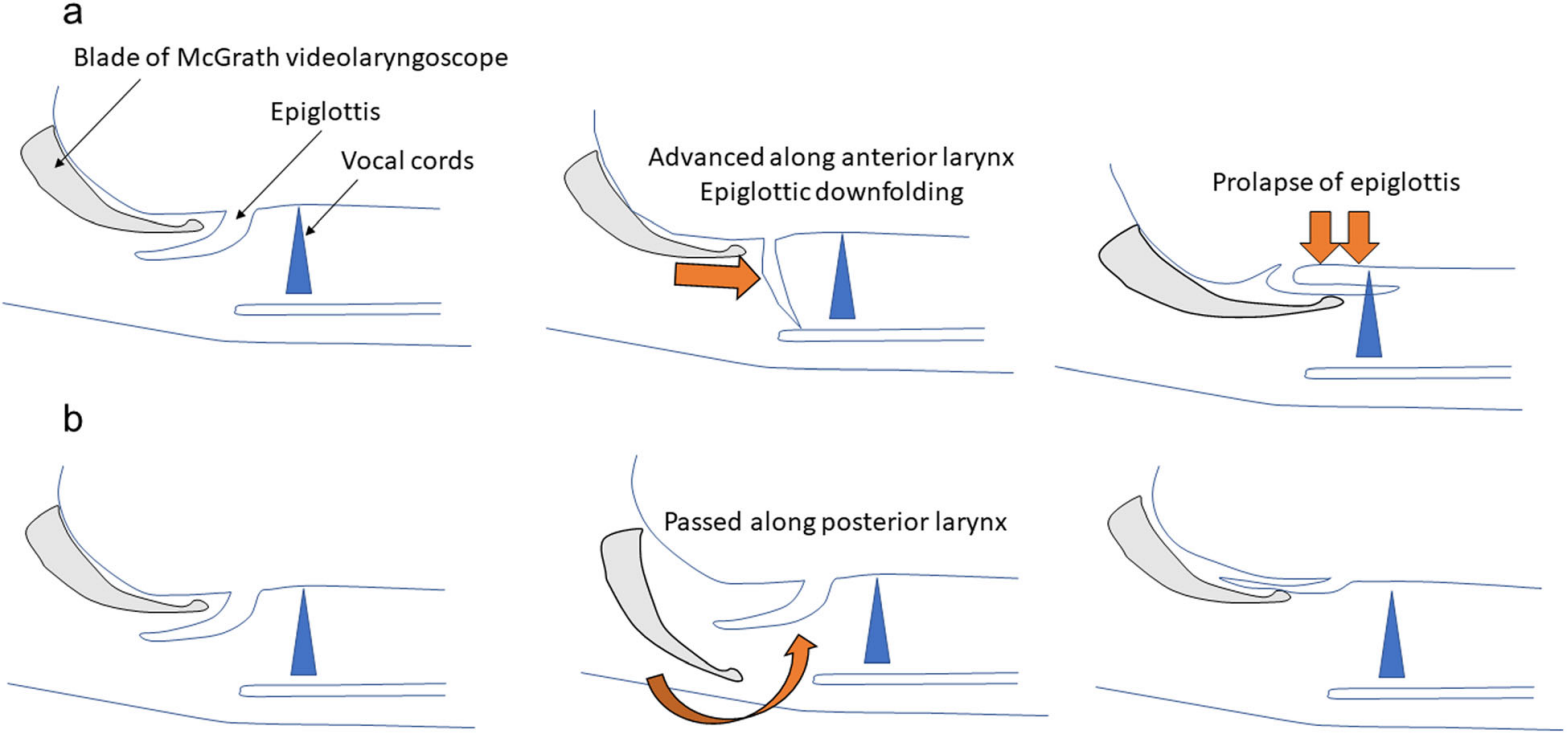
Diagram showing the mechanism of epiglottic downfolding and how this complication can be avoided. **a** When the blade of the McGrath videolaryngoscope is passed along the anterior larynx to directly elevate the epiglottis, epiglottic prolapse can occur. However, this could be difficult to be detected because the epiglottis is not visible on the screen of the McGrath videolaryngoscope. **b** If it is necessary to elevate the epiglottis directly, the epiglottis should be lifted from the dorsal side to prevent downfolding

Epiglottic downfolding into the laryngeal inlet has been reported to be associated with blind intubation techniques such as supraglottic airway-guided intubation [3], fiberoptic intubation [4], and light-guided intubation [5, 6] because these techniques do not enable observation of the epiglottis during intubation. However, it can also occur using the Macintosh laryngoscope [1, 2] or the Pentax-AWS which provide better visualization [7]. A previous case series [7] reported that the rate of epiglottis prolapse with the Pentax-AWS was approximately three in 1000 intubations. In these cases, prolapse was caused by the blade of the Pentax-AWS as it passed along the anterior larynx. To prevent this complication, the authors recommended that the Pentax-AWS blade should be advanced along the posterior aspect of the airway as in the laryngeal mask airway insertion technique [7]. Epiglottic downfolding into the laryngeal inlet when using the McGrath videolaryngoscope may be caused by the same mechanism as for the Pentax-AWS (Fig. 2a). Although the typical insertion technique for the McGrath videolaryngoscope differs from that of the AWS in that the tip of the blade is usually inserted into the vallecula, as for the Macintosh laryngoscope, the blade can also be used to elevate the epiglottis directly. When it is necessary to elevate the epiglottis directly, we recommend that the blade should be advanced along the posterior aspect of the airway; in other words, the epiglottis should be lifted from the dorsal side to prevent downfolding (Fig. 2b).

There are no established methods to correct epiglottis prolapse. In previous cases, the epiglottis has been restored to its original position using a McCoy-type laryngoscope [3], a fiberscope [4], endoscopic forceps [6], and partial extubation [1, 2, 5, 7]. In the present case report, the epiglottis prolapse was corrected easily by gentle glottic exposure using the McGrath videolaryngoscope without extubation or the use of any specialized devices. In this case, early detection was a possible reason for success. The problem was easily corrected because the epiglottis was still soft and elastic at the time of detection. If detection is delayed, the epiglottis can become edematous and, thus, more difficult to be restored. Therefore, whenever possible, anesthesiologists should carefully observe the condition of the epiglottis during and immediately after tracheal intubation using the McGrath videolaryngoscope.

In this report, we described a case of epiglottic downfolding into the laryngeal inlet after tracheal intubation using the McGrath videolaryngoscope. After the prolapse, we were able to restore the epiglottis to its original position by gentle glottic exposure using the McGrath videolaryngoscope without removal of the endotracheal tube. During tracheal intubation using the McGrath videolaryngoscope, Macintosh-type glottic exposure and careful observation of the epiglottis can enable the prevention and detection of epiglottis prolapse.

## Data Availability

Not applicable

## Abbreviations

McGrath: McGRATH^TM^ MAC
Pentax-AWS: Pentax Airway Scope®
